# Tumor Profiling at the Service of Cancer Therapy

**DOI:** 10.3389/fonc.2020.595613

**Published:** 2021-01-11

**Authors:** Ceres Fernandez-Rozadilla, Ana Rita Simões, Matilde E. Lleonart, Amancio Carnero, Ángel Carracedo

**Affiliations:** ^1^Grupo de Medicina Xenómica (USC), Instituto de Investigación Sanitaria de Santiago (IDIS), Santiago de Compostela, Spain; ^2^Biomedical Research in Cancer Stem Cells, Vall d´Hebron Research Institute (VHIR), Barcelona, Spain; ^3^Spanish Biomedical Research Network Centre in Oncology, CIBERONC, Madrid, Spain; ^4^Instituto de Biomedicina de Sevilla, IBIS, Hospital Universitario Virgen del Rocío, Universidad de Sevilla, Consejo Superior de Investigaciones Científicas, Seville, Spain; ^5^Grupo de Medicina Xenómica (USC), Fundación Pública Galega de Medicina Xenómica, Santiago de Compostela, Spain

**Keywords:** cancer treatment, omics, profiling, immunotherapy, personalized medicine, precision oncology

## Abstract

Cancer treatment options have evolved significantly in the past few years. From the initial surgical procedures, to the latest next-generation technologies, we are now in the position to analyze and understand tumors in a one-by-one basis and use that to our advantage to provide with individualized treatment options that may increase patient survival. In this review, we will focus on how tumor profiling has evolved over the past decades to deliver more efficient and personalized treatment options, and how novel technologies can help us envisage the future of precision oncology toward a better management and, ultimately, increased survival.

## Introduction

Classically, choice of therapy depends mainly on location and grade of the primary tumor (as ascertained by histology), as well as the stage of the disease. Types of therapies are classically subdivided into surgery, chemotherapy, radiotherapy, hormonal therapy and immunotherapy, although the boundaries between these categories are sometimes blurry. Typically, localized tumors will be selected for resection *via* surgical procedures, which may be coupled with preceding (neoadjuvant) therapy to shrink the tumor prior to removal, and/or followed by adjuvant therapy to reduce the chances of relapse.

Cancer treatments have suffered a considerable revolution in the past few years owing to the recent development of high-throughput omic technologies. These have constituted the flourishing of targeted therapies, which can drive the final hurdle from histologic treatments to individualized treatments that attack each tumor precisely based on its very own molecular features. In this review, we summarize the road so far, from the earliest treatments to current strategies and what lies beyond.

## Cancer Therapy

**Figure 1:**
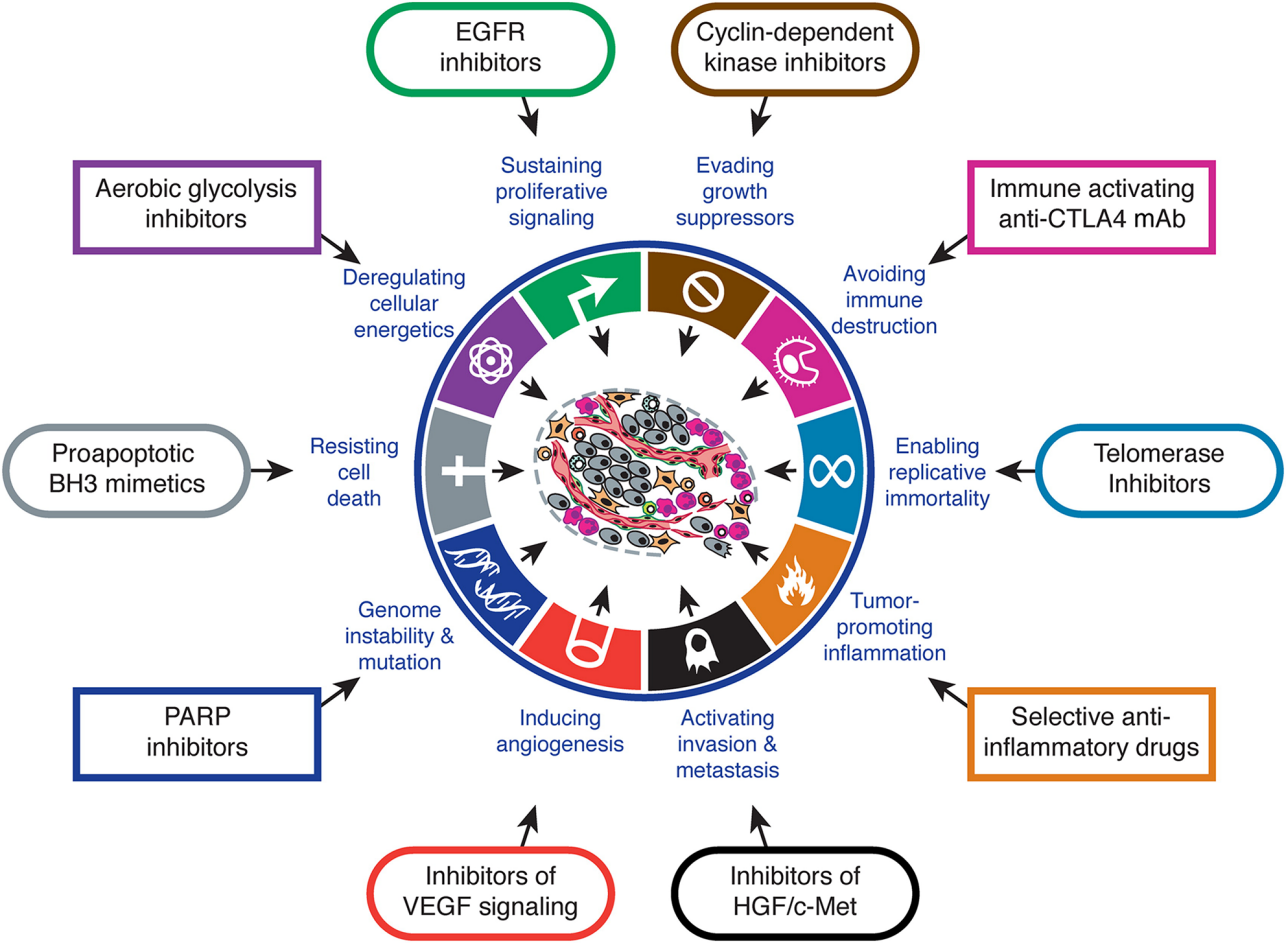
Hallmarks of cancer and therapeutic implications. Adapted from (3).

**Figure 2:**
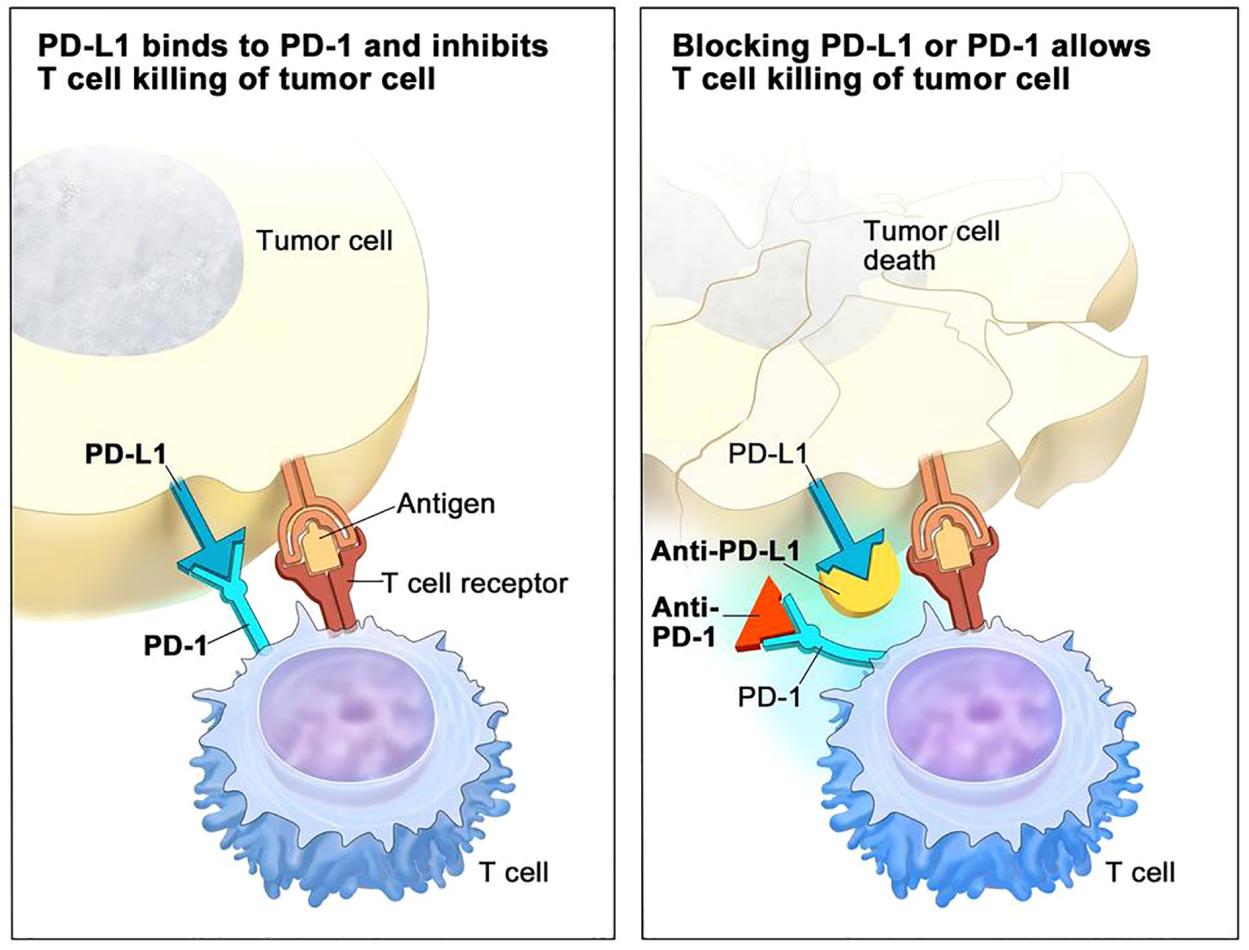
PD-1/PDL-1 immune checkpoint inhibitor (ICI) mechanisms of action in immunotherapy (adapted from NCI – www.cancer.gov).

**Figure 3:**
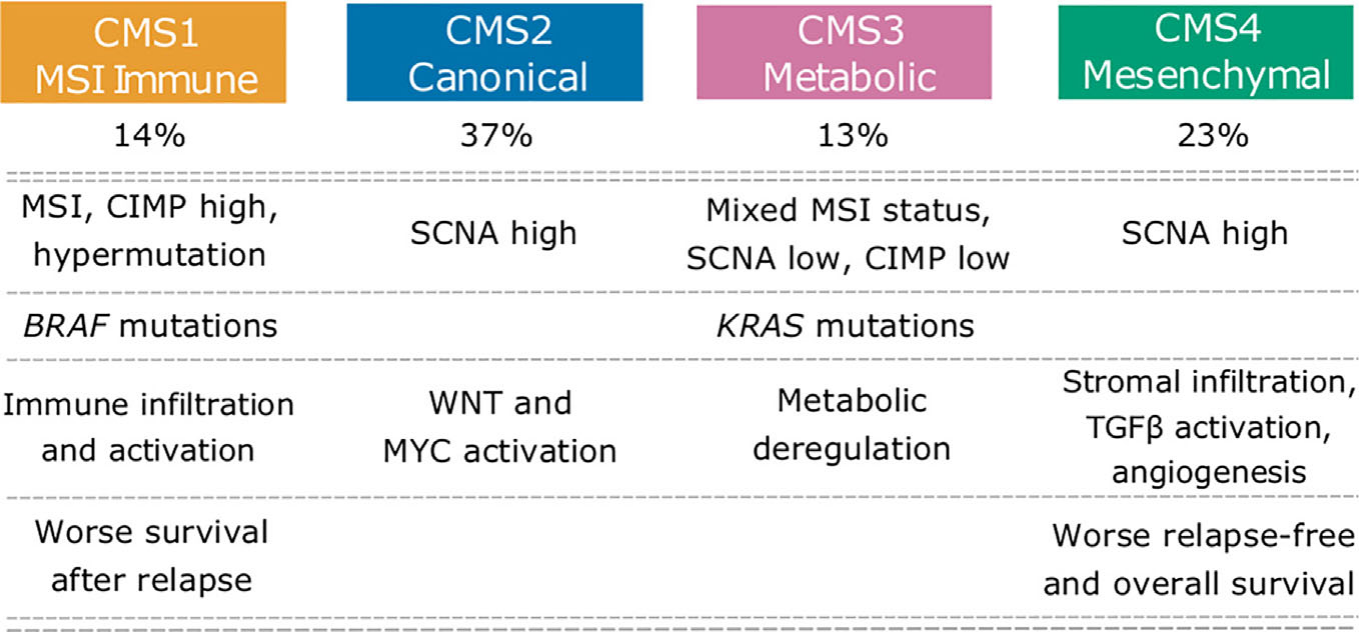
Colorectal cancer consensus molecular subtypes (CMS). CMS showing their main molecular features. Adapted from (105). This subtyping has influence on choice of therapeutic strategy and also has prognostic value at the therapeutic level. CMS1 cancers have an indication to be treated with immunotherapy and better prognosis than CMS2-4.

It has been known for many years now that cancer cells have particular features that make them different to normal cells of the same tissue. Arguably, one of the most remarkable ones is the fact that tumor cells can obtain their energy through glycolysis instead of oxidative phosphorilation, even in the presence of oxygen. This feature is known as the *Warburg effect* after the clinician who discovered it (1), and is one of the biological capabilities acquired during the multistep development of human tumors, also known as the *hallmarks of cancer* (2, 3). The Warburg effect has meaningful implications in cancer cell metabolism, thereby allowing these cells to gain a selective advantage when competing for shared and limited energy resources, which results in them proliferating more rapidly (4). The same happens to the other features, which must occur at a certain point in time over the course of cancer development. Hence, targeting one or several of these features is key for cancer therapeutic intervention [**Figure 1** - (3)].

Cancer therapeutic approaches were initially based on surgical removal of the tumor, with radiotherapy moving on quickly in the early 1900s (5). By the 1930s, the field was starting to point toward novel strategies, based on the findings on tumor biology provided by Warburg himself and others, and by the 1930s, Paul Ehrlich who coined the term “chemotherapy” to describe the use of chemical compounds to fight cancer. By 1946, the first alkylating agent was approved as a chemotherapeutic agent (6), and since then, several other agents have been used in the fight against cancer. The common feature that all of these chemotherapeutics share is their use of these particular properties of cancer cells to destroy them. For instance, alkylating agents such as the platins (carboplatin, cisplatin, and oxaliplatin) and topoisomerase inhibitors like irinotecan produce DNA damage; alkaloids such as paclitaxel and docetaxel disrupt cell division and, antimetabolites like 5-fluorouracil, gemcitabine or methotrexate work by inhibiting cell division (7–9) (**Table 1**).

**Table 1:** Commonly used chemotherapeutic agents.

Types of chemotherapy	Subtype	Examples
Alkylating agents	Oxazsaphosphorines	cyclophosphamide, ifosfamide
	Nitrogen mustards	busulfan, chlorambucil, melphalan
	Hydrazine	temozolomide
	Platinum-based agents	cisplatin, carboplatin, oxaliplatin
Antimetabolites	Pyrimidine antagonists	cytarabine, 5-fluorouracil, gemcitabine, capecitabine
	Purine antagonists	fludarabine
	Purine analogs	6-mercaptopurine, azathioprine, cladribine
	Antifolates	methotrexate, pemetrexed, pralatrexate
	Ribonucleotide reductase inhibitors	hydroxyurea
Topoisomerase inhibitors	Topoisomerase I inhibitors	irinotecan, topotecan
	Topoisomerase II inhibitors	etoposide; teniposide; anthracyclines, e.g., idarubicin, daunorubicin, doxorubicin
Mitotic spindle inhibitors	Taxanes	docetaxel, paclitaxel
	Vinca alkaloids	vincristine, vinblastine
Other	Enzymes	l-asparaginase
	Tyrosine kinase inhibitors	imatinib and erlotinib
	Antibiotics	bleomycin, actinomycin D, anthracyclines
	Proteasome inhibitors	bortezomib
	Autophagy inhibitors	hydroxychloroquine

In general, traditional chemotherapeutic agents are mainly cytotoxic (also coined cytostatic), which means they interfere with and stop cell division. This is primarily aimed to target highly-proliferating cells, such as neoplastic ones. Cytotoxic agents may be used alone (monotherapy) or in combination with other therapies, and up to today, still constitute the backbone of cancer treatment (10). Nevertheless, there are two main problems with cytotoxic therapies: response (or sometimes resistance) and toxicity. For the former, response rates to standard cytotoxic chemotherapy are varied and depend greatly on tumor site and stage. For instance, it is well known that advanced pancreatic tumors only present response rates of about 20% to classical treatments with gemcitabine and nab-paclitaxel (11, 12). Moreover, the development of secondary resistance (refractory response after an initial responsive period) is also common, and is one of the major causes of failure of cancer treatment. For the latter, the fact that cytotoxic agents target rapidly dividing cells may also affect other normal cell types, such as the bone marrow, hair follicles or digestive tract, thereby resulting in the development of adverse drug reactions that may result in discontinuation in the administration of the drug, and therefore, may compromise its curative purpose (13, 14).

## Targeted Therapies

### Small Molecules

Regardless of all hallmarks acquired by tumor cells, cancer is ultimately a genetic disease caused by genomic mutations in genes that allow them to obtain a selective advantage, whether that is in terms of faster proliferation, nutrient acquisition or blood vessel formation (15). Research on oncogenes and tumor suppressor genes (the two main types of genes in which mutations drive cancer development) has been strongly active since the first description of a cancer-causing mutation (16–18).

Generic cytotoxic drugs are not enough to target these changes specifically, and therefore the identification of these driver mutational events launched for the first time the possibility to test for and treat against specific mutations appearing in particular genes and tumors, and hence, provided the basis for targeted cancer therapies. Indeed, it has been described that the Warburg effect is possibly an early event in oncogenesis that is an immediate consequence of an initial oncogenic mutation, such as that of *KRAS* in pancreatic cancer or *BRAF* in melanoma, and may occur in early stage lesions as well (19, 20).

Targeted therapies work primarily by attacking deregulated proteins that support survival of cancer cells (21, 22). There is quite a variety of small molecules to target these proteins, but arguably, kinase inhibitors have been the most successful. We know that in many tumors, signaling pathways regulated by protein kinases are the frequent targets of somatic mutations, and indeed of the more than 100 oncogenes known, many encode kinases (23). These kinases may be led to aberrant function by several mutational processes, including genomic rearrangements, gain-of-function mutations, or overexpression and/or gene amplification, which ultimately result in the loss of regulatory constraints and a constitutive activation of the protein.

The first targeted therapy directed against a specific genetic abnormality was imatinib, a tyrosin-kinase inhibitor (TKI) that inhibits proliferation of BCR-ABL-expressing hematopoietic cells by specifically targeting the constitutively active fusion protein produced by the reciprocal translocation of chromosomes 9 and 22 (t(9;22)(q34;q11)) (24, 25). The list of targeted therapies has rapidly expanded ever since its discovery, and a selection of the most commonly used targeted therapies and their corresponding molecular changes is represented on **Table 2**. Albeit the explosion of targeted therapies, these small-molecule approaches have been more favorable for cancers like lung, colorectal, breast, lymphoma and leukemia, as they focus on particular molecular changes unique to a specific cancer, whereas other cancer types such as pancreatic or upper gastrointestinal tumors have experienced less progress in targeted drug therapy development.

**Table 2:** List of common small molecule therapies (26–29).

Target	Drug	Tumor type
BCR-ABL	imatinib; dasatinib; nilotinib; bosutinib; regorafenib; ponatinib	CML; ALL; GIST; CRC
PDGFR	imatinib; dasatinib; nilotinib; sunitinib; sorafenib; regorafenib; erdafitinib; lenvatinib; pazopanib	ALL; CML; GIST; RCC; pNET; HCC; thyroid cancer; CRC; UC; RCC; soft tissue sarcoma
EGFR	afatinib; gefitinib; osimertinib; vandetanib; erlotinib; lapatinib; dacomitinib; neratinib	NSCLC; PDAC; medullary thyroid cancer; BrCA
FGFR	erdafitinib; lenvatinib; pazopanib	UC; thyroid cancer, HCC; RCC; soft tissue sarcoma
HER	afatinib; osimertinib; neratinib; lapatinib	NSCLC; BrCA
CDK 4/6	ribociclib; abemaciclib; palbociclib	BrCA
C-KIT	imatinib; dasatinib; nilotinib; sunitinib; sorafenib; regorafenib; erdafitinib; lenvatinib; cabozantinib; pazopanib	CML; ALL; GIST; HCC; pNET; RCC; thyroid cancer; CRC; UC; soft tissue sarcoma
SCF	imatinib	CML; ALL; GIST
SRC	dasatinib: bosutinib; vandetanib	ALL; CML; medullary thyroid cancer
CSF	nilotinib; sunitinib; erdafitinib	CML; GIST; RCC; pNET; UC
DDR	nilotinib; regorafenib	CML; CRC
C-MET	crizotinib; cabozantinib	NSCLC; HCC; RCC
VEGFR	sunitinib; sorafenib axitinib;, vandetanib; regorafenib; erdafitinib; lenvatinib; cabozantinib; pazopanib	RCC; HCC; medullary thyroid cancer; GIST; pNET; thyroid cancer; CRC; UC; soft tissue sarcoma
RET	vandetanib; sunitinib; regorafenib; sorafenib; erdafitinib; alectinib; lenvatinib; cabozantinib	Medullary thyroid cancer; GIST; RCC; pNET; CRC; HCC; thyroid cancer; UC; NSCLC
TIE2	vandetanib; regorafenib; cabozantinib	Medullary thyroid cancer; CRC; RCC; HCC
RAF	vemurafenib; sorafenib; regorafenib; encorafenib; dabrafenib	Melanoma; HCC; RCC; thyroid cancer; CRC
PARP	olaparib; rucaparib; talazoparib; niraparib	ovarian cancer; BrCA
TRK	larotrectinib; regorafenib; entrectinib; cabozantinib; lorlatinib	solid tumors; CRC; NSCLC; HCC; RCC
BTK	ibrutinib	MCL; CLL; SLL
MEK	cobimetinib; binimetinib; trametinib	melanoma
FTL	sorafenib; sunitinib; erdafitinib; brigatinib; cabozantinib; gilteritinib	HCC; RCC; thyroid cancer; GIST; pNET; UC; NSCLC; AML
ROS1	entrectinib; crizotinib; brigatinib; lorlatinib; ceritinib; cabozantinib	solid tumors; NSCLC; RCC; HCC
ALK	entrectinib; alectinib; crizotinib; brigatinib; lorlatinib; ceritinib	solid tumors; NSCLC
IGF-1R	brigatinib; ceritinib	NSCLC
IDH1	ivosidenib; enasidenib	AML
26S proteasome	bortezomib; carfilzomib; marizomib	multiple myeloma; MCL
PI3KCA	alpelisib	BrCA
PI3K	duvelisib; copanlisib	CLL, SLL; Follicular lymphoma

CML, chronic myelogenous leukemia; ALL, acute lymphoblastic leukemia; pNET, pancreatic neuroendocrine tumors; NSCLC, non-small-cell lung cancer; PDAC, Pancreatic ductal adenocarcinoma; UC, urothelial cancer; HCC, hepatocellular carcinoma; RCC, renal cell carcinoma; MCL, Mantle cell lymphoma; CLL, Chronic lymphocytic leukemia; SLL, Small lymphocytic lymphoma; AML, acute myeloid leukemia; BrCA, breast cancer; CRC, colorectal cancer.

### Immunomodulation and Immunotherapy

Another important hallmark of cancer is that, for a tumor to arise, it must evade the strict control to which malfunctioning cells are subject by the immune system (3). Although it is still unclear whether this immune evasion happens as a passive or active process (or possibly even both), it is however certain that at some or other point tumors acquire the ability to surpass the control of the immune system. This observation gave rise to the field that utilizes the artificial stimulation of the immune system to treat cancer: immunotherapy. Immunotherapy has become such an important part of cancer therapy in the past few decades that it was merited with the Nobel Prize on Physiology or Medicine to James P. Allison and Tasuku Honjo for their discovery of cancer therapy by inhibition of negative immune regulation.

There are two main types of immunotherapies: passive immunotherapy, which consists in the blocking of cell surface receptors that are specific to tumor cells, and active immunotherapy, that aims to stimulate the patient´s immune system to reactivate the fight against cancer cells (30, 31). For the former, monoclonal antibodies (mAbs) have been the main strategy. These antibodies are produced specifically to block cell surface receptors that are present (ideally) exclusively on tumor cells and tumor-promoting molecules. They recognize a tumor antigen and cause cell death through various mechanisms, including apoptosis or indirect elimination by recruitment of immune cells with cytotoxic properties, or by activation of the complement cascade. Examples of these mAbs are those directed toward the vascular endothelial growth factor (VEGF), interleukins or the macrophage migration inhibitory factor (MIF) (31–34).

A special type of mAbs that has claimed great benefits in patient survival over the past ten years are immune checkpoint inhibitors (ICIs) (35). Their success radicates in the fact that may be directed to the tumor cells but also to T cells, to reinstate recognition of tumor cells by the immune system, thereby relaunching an immune response. The main three ICIs used to data have been CTLA-4, PD-1 and PD-L1 inhibitors (36) (**Figure 2** - www.cancer.gov).

As for small molecules, immunotherapy has also become an important tool in the development of targeted anticancer therapies, and there are nowadays numerous mAbs to treat various types of cancers, with numbers rapidly increasing (**Table 3**). Among these, rituximab (anti-CD20 mAb used in the treatment of non-Hodgkin lymphoma) is possibly the most extensively used. However, over 2900 clinical trials have been reported on the use of mAbs in cancer, and many others are currently ongoing in cancer patients (ClinicalTrials.gov).

**Table 3:** List of common monoclonal antibody (mAb) therapies (27, 37–40).

Target	Drug	Tumor type
HER2	adotrastuzumab; trastuzumab; pertuzumab	BrCA
EGFR	cetuximab; panitumumab; necitumumab	CRC, HNSCC; NSCLC; PDAC; glioma; Squamous NSCLC
VEGFR	ramucirumab	gastric cancer; NSCLC
VEGF	bevacizumab	CRC; NSCLC; BrCA; Glioblastoma; RCC
CD-20	rituximab; ofatumumab; ibritumomab; tositumomab; obinutuzumab	Non-Hodgkin lymphoma; CLL; follicular lymphoma
CD-22	inotuzumab	ALL
CD-52	alemtuzumab	CLL
CD-33	gemtuzumab	AML
CD-30	brentuximab	Hodgkin lymphoma; anaplastic large cell lymphoma
CD19/CD3	blinatumomab	ALL
CD38	daratumumab	multiple myeloma
CTLA-4	ipilimumab	melanoma; RCC
PD-1	nivolumab	melanoma; NSCLC; SCLC; RCC; UC; Hodgkin lymphoma; HNSCC; MSI-H/dMMR CRC; HCC
PD-L1	atezolizumab; avelumab; cemiplimab; pembrolizumab; durvalumab	UC; NSCLC; BrCA; RCC; CSCC; melanoma; NSCLC; HNSCC; Hodgkin lymphoma; MSI-H cancer; gastric cancer; cervical cancer; HCC; MCC
RANKL	denosumab	giant cell tumor of the bone
GD2	dinutuximab	pediatric neuroblastoma
PDGFR	olaratumab	soft tissue sarcoma
SLAMF7	elotuzumab	multiple myeloma

BrCA, breast cancer; CRC, colorectal cancer; HNSCC, Head and neck squamous cell carcinoma; NSCLC, non-small-cell lung cancer; PDAC, Pancreatic ductal adenocarcinoma; RCC, renal cell carcinoma; CLL, chronic lymphocytic leukemia; ALL, acute lymphoblastic leukemia; AML, acute myeloid leukemia; UC, urothelial cancer; MCL, Mantle cell lymphoma; MSI-H, microsatellite instability-high; dMMR, mismatch repair deficient; MCC, Merkel cell carcinoma; CSCC, cutaneous squamous cell carcinoma.

Active immunotherapy is composed of CAR-T antibodies, which are harvested, modified T cells from the patient that are genetically altered to specifically recognize cancer cells when infused back into the patient. The features and implications of CAR-T technologies are extensive and far beyond the scope of this review, but comprehensive reviews can be found in (41–44).

### Molecular Testing for Targeted Therapies

Because targeted therapies are particularly directed toward the specific changes present in a given tumor’s cells, clinical molecular pathology analysis has therefore become an indispensable laboratory tool that can be used to characterize tumor biology and to drive therapeutic decisions (45). This is known as pharmacodiagnostics and aims to determine whether a patient will successfully respond to a given therapy, and is therefore an intrinsic part of personalized medicine approaches. The indications for molecular testing in the most prevalent tumor types are summarized in **Table 4**.

**Table 4:** Molecular testing for each tumor type (46–48).

Tumor type	Gene	Change	Treatment	Drugs (examples)
NSCLC	EGFR	mutation	TKI	gefinitib; erlotinib; afatinib, dacomitinib; osimertinib
	ALK	translocation	TKI	crizotinib, ceritinib, alectinib, lorlatinib, brigatinib
	ROS1	translocation	TKI	crizotinib, entrectinib
	PD-L1	protein expression	PD-1 blocking antibody	pembrolizumab
	KRAS	mutation	TKIs	
	BRAF	mutation	kinase inhibitors	vemurafenib; dabrafenib
	HER2	mutation	kinase inhibitors	afatinib; osimertinib
	MET	amplification, mutation	kinase inhibitors	crizotinib; cabozantinib
	RET	fusion, rearrangement	kinase inhibitors	alectinib
Melanoma	BRAF	mutation	kinase inhibitors	dabrafenib; trametinib, vemurafenib; cobimetinib, encorafenib; binimetinib
	KIT	mutation	kinase inhibitor	imatinib
GIST	KIT	mutation	kinase inhibitors	imatinib
	PDGFR	mutation	kinase inhibitors	imatinib
	HER2	gene amplification	HER receptor antagonists	trastuzumab
	PD-L1	expression	PD-1 blocking antibody	pembrolizumab
Pancreatic cancer	BRCA1/2	mutation	PARP-inhibitors	olaparib
CRC	KRAS/NRAS	mutation	EGFR antagonists	cetuximab; panitumumab
	BRAF	mutation	EGFR antagonist	cetuximab; panitumumab
	MSI-H or dMMR	expression	PD-1 blocking antibody	nivolumab; ipilimumab
BrCa	HER2	amplification	HER2‐targeted therapy	trastuzumab; lapatinib; pertuzumab
	BRCA1/2	mutation	PARP inhibitors	olaparib, talazoparib, rucaparib
	PI3KCA	mutation	kinase inhibitors	alpelisib
OvCa	BRCA1/2	mutation	PARP-inhibitors	olaparib, talazoparib, rucaparib
	ATM			
	BRiP1			
	CHEK2			
	PALB2			
	RAD51C			
	RAD51D			
Sarcoma	MDM2, CDK4	amplification	CDK4/CDK6 inhibitors	palbociclib
	IDH1/IDH2	mutation	IDH1 inhibitor	ivosidenib
Melanoma	BRAF	mutation	kinase inhibitors	vemurafenib; encorafenib; dabrafenib
	KIT	mutation	kinase inhibitors	imatinib; nilotinib
Head and Neck Cancer	PD-L1	protein expression	PD-1-blocking antibody	pembrolizumab
Solid tumors	MMR/MSI	expression	PD-1-blocking antibody	pembrolizumab
	TRK	fusion	kinase inhibitors	entrectinib; larotrectinib
Chronic myeloid leukemia	BCR/ABL	fusion	kinase inhibitors	imatinib, dasatinib, nilotinib, bosutinib, ponatinib
	PI3K	mutation	kinase inhibitors	duvelisib
Acute myeloid leukemia	IDH1/2	mutation	IDH1 inhibitors	ivosidenib, enasidenib
	FLT3	mutation	kinase inhibitors	gilteritinib
Follicular lymphoma	PI3K	mutation	kinase inhibitors	copanlisib
Urothelial cancer	FGFR2/3	mutation, fusion	kinase inhibitors	erdafitinib

List of molecular tests currently indicated for the most prevalent tumor types.

Classical detection methods in cancer pathology include gold standard techniques in molecular biology: immunohistochemistry (IHC): as for the case of p16 staining for HPV infection in FFPE tissues (49); fluorescent in-situ hybridisation, (FISH) to detect chromosomal rearrangements in hematological malignancies (50, 51), PCR or Sanger sequencing for point mutations (52).

These tests are currently essential to classify tumors and decide on treatment strategies. For example, breast tumor biology has historically been classified based on immunohistochemical (IHC) staining of proliferation proteins (Ki-67), hormone receptor status (estrogen receptor alpha (ER), progesterone receptor (PR) and/or androgen receptor (AR), and the presence/absence of specific cytokeratins (CK). Therapeutic strategies are based on this histological classification and Ki-67 assays have additional prognostic value (53).

## Tumor Profiling to Guide Cancer Therapy

Targeted therapies provided the first evidence that treating a tumor based on its molecular features could result in better patient outcome in terms of increased survival. However, molecular testing based on the features provided on **Table 4** is clearly insufficient, particularly for underrepresented tumors that tend to have worse prognosis, such as pancreatic or endometrial cancers. Therefore, there have been extensive efforts to upgrade our molecular knowledge on cancer to a more comprehensive view of each individual cancer. Tumor profiling constitutes the pinnacle of these efforts, where we aim to classify neoplasms into subgroups that give us information about how the cancer has evolved, how it can be better treated, and how we should direct drug design strategies to treat them. Several approaches to tumor profiling have been undertaken in the past few years that will be discussed below.

### Genomics

The publication of the human genome sequence in 2003 (54) and the implementation of next-generation sequencing technologies since the turn of the century has allowed for our knowledge on germline and somatic tumor genomics to increase tremendously in the past two decades. This is particularly relevant in the context of targeted cancer treatments, where we aim to achieve better outcomes by treating tumors with drugs that are specifically matched to their molecular features. Whole-exome and whole-genome cancer sequencing initiatives like The Cancer Genome Atlas (TGGA) (cancergenome.nih.gov/) and the International Cancer Genome Consortium (icgc.org) have sequenced hundreds of cancers across 38 tumor types to provide the most comprehensive cancer genome database to date (55).

The benefits of these initiatives have been unprecedented and multiple. Firstly, we have been able to identify a much larger proportion of cancer driver mutations. These are changes that give the tumor cell a selective advantage in its microenvironment, through either increasing its survival or reproduction. Driver mutations tend to cause clonal expansions and are the fundamental first step of cancer development. Therefore, identifying them is key for the design of targeted therapies that can stop cancer growth and spreading. Driver mutations happen preferentially in oncogenes and tumor suppressor genes, and hence the list of cancer genes has increased exponentially in the past few years (56–59). Moreover, because whole-genome cancer sequencing provides a more comprehensive assessment of the mutational spectrum, we can assess not only point mutations on a large scale, but also other genomic features that can be relevant drivers, such as mutations in non-coding regions (60–62), CNVs and structural variations (63).

Secondly, because cancer is highly heritable (64), candidate driver genes may also be identified by NGS of the patient´s germline DNA following Knudson´s two hit hypothesis (18). Since somatic mutation analysis inherently requires the sequencing of the matching normal tissue, this can be used to advance into the description of germline variants that confer cancer predisposition (65). Germline pathogenic mutational events may have important consequences for cancer treatment, as it has been proven that both germline and somatic mutations in the homologous recombination genes *BRCA1*, *BRCA2*, *PALB2* (also termed “BRCAness”) respond well to treatment with poly-ADP ribose polymerase (PARP) inhibitors. This is true for several cancer types, including breast, ovarian, prostate and pancreatic tumors (66, 67). Another example is that of tumors arising from germline and/or somatic mutations in polymerases ϵ and δ (*POLE* and *POLD1* genes), which have an indication for treatment with immunotherapy (68).

In any case, the determination of both germline and somatic mutation events leading up to cancer has great consequence for the establishment of actionable mutations. A study performed on 2,520 pairs of primary and metastatic tissue tumors found that 62% of patients presented with genetic variants that could be used to stratify patients toward either approved therapies or those in clinical trials (69). Moreover, half of the patients with a predicted candidate actionable event (31% of total) contained a biomarker with a predicted sensitivity to a drug at level A (approved anti-cancer drugs) and lacked any known resistance biomarkers for the same drug. Hence, big efforts are being made at current to categorize somatic mutation variants into likely actionable mutations in order to advance in the design of novel anticancer drugs (70, 71).

All of these key features of genomic high-throughput sequencing are ultimately key for tumor profiling, and have made it possible to gain a much better insight into the molecular and genomic features of different tumor types. This has been particularly relevant for those with less available therapeutic options (72, 73). Relevant developments in novel targeted therapies that have sprung thereof are for instance the treatment of tumors with *ARID1A* mutations and dasatinib (74, 75); among many others.

### Epigenomics

Carcinogenesis has been shown to be accompanied by widespread DNA methylation changes in the tumor cell, that are usually visible as a globally hypomethylated genome mimicking a stem cell phenotype (76). These changes in the methylation patterns of tumor cells can appear as a result of genomic mutations or constitute neoplastic drivers by themselves (77). For instance, the germline or somatic methylation of the *MLH1* gene promoter is a well-known epigenetic driver event. Many of these epigenetic changes occur early in tumorigenesis and are highly pervasive across a tumor type. Therefore, intensive studies have been performed to elucidate the methylatory landscape of several cancer types, including breast (78), lung (79), prostate (80), or CLL (81), among others (82).

Interestingly, for CRC, a redistribution of methylation sites has been observed, where there is focal hypermethylation of CpG islands on tumor suppressor genes. This phenomenon is called the CpG island methylator phenotype (CIMP) (83), and it has since been discovered in multiple other tumor types, including bladder, breast, gastric or pancreatic cancers (84–88). The CIMP phenotype has been linked to multiple genetic causes, including at least the *BRAF* V600E mutation, or pathogenic mutations in the *IDH1* gene (89, 90).

Because epigenetic alterations are reversible, they can be a substrate for therapy development as well as influence the choice of treatment. DNA methylation differences have been observed, for instance, between radio-sensitive and radio-resistant cell lines (91). Moreover, methylation of the *MGMT* promoter in gliomas is a useful predictor of the responsiveness of the tumors to alkylating agents (92). Differential methylation has also been associated with increased risk of recurrence in NSCLC and breast cancers (93, 94), and methylation of a CpG in the transcription factor *FOXP1* is predictive of response to ICB in NSCLC patients (95). In the case of CIMP tumors, it has been noticed that it almost invariably results in hypermethylation of the *MLH1* promoter, which in turn provokes a MSI phenotype, and has been shown to correlate with response to immune checkpoint inhibitors (88, 96).

Nevertheless, data about the relationship between drug response and the epigenomic variations is still scarce mainly because the epigenome is highly variable between individuals, and hence therapeutic choice based on methylation profiling is rare. Alternatively, other epigenomic events (histone modifications, chromosome remodeling or RNA editing) have also been explored to a smaller extent that could also potentially identify druggable pathways (97).

### Transcriptomics

The study of the genome provides only a steady‐state view of highly dynamic molecular populations, and the information reflected in the genome may not be relevant if, for instance, the gene product is not expressed in the tissue-of-origin of the tumor. RNA sequencing (RNA-seq) has also been a relevant source of knowledge to inform on tumor profiling and therapeutic management. As sequencing methods became more cost-efficient, there have been large-scale molecular profiling efforts that have inspected RNA-seq in tumors (TCGA) and in normal tissues [the Genotype-Tissue Expression project – GTEx – (98)]. Transcriptome profiling presents several advantages over genomics and epigenomics studies: first, it is the most reliable way to detect gene fusions *ad hoc* and at a great scale, which may be particularly relevant in some types of tumors (99); secondly, gene expression signatures can be derived to infer prognostic and predictive information, and they allow for refinement of disease subclassification beyond what can be achieved by currently validated biomarkers (100); thirdly, it can give us information not only from the tumor, but also from the microenvironment, including cell composition derivations using deconvolution strategies [and these may be especially relevant for targeted immunotherapy strategies (101)]; fourth, gene expression analyses can be done reliably on a single-cell basis, to target, for instance, activated pathways in cancer stem cells (102). Lastly, transcriptome quantification can summarize the effects of known and unknown driver (epi)genomic events into measurable phenotypes, and therefore, it has the potential to link tumor genotypes to their phenotypic consequences (103).

Hence, transcriptomics has been essential in the road toward a more efficient tumor molecular profiling. There are now four fully established CRC consensus molecular subtypes (CMS) based on transcriptomic profiling: CMS1 (MSI), CMS2 (canonical), CMS3 (metabolic) and CMS4 (mesenchymal). Given the consistent classification, it would be then advisable to devise therapeutic strategies based on these molecular profiles, and indeed, CMS1 tumors are indicated to receive immunotherapy with ICIs, such as pembrolizumab or nivolumab. For the other categories however, studies are still underway to design novel targeted treatment options (104) [**Figure 3** - (105)].

**Figure 4:**
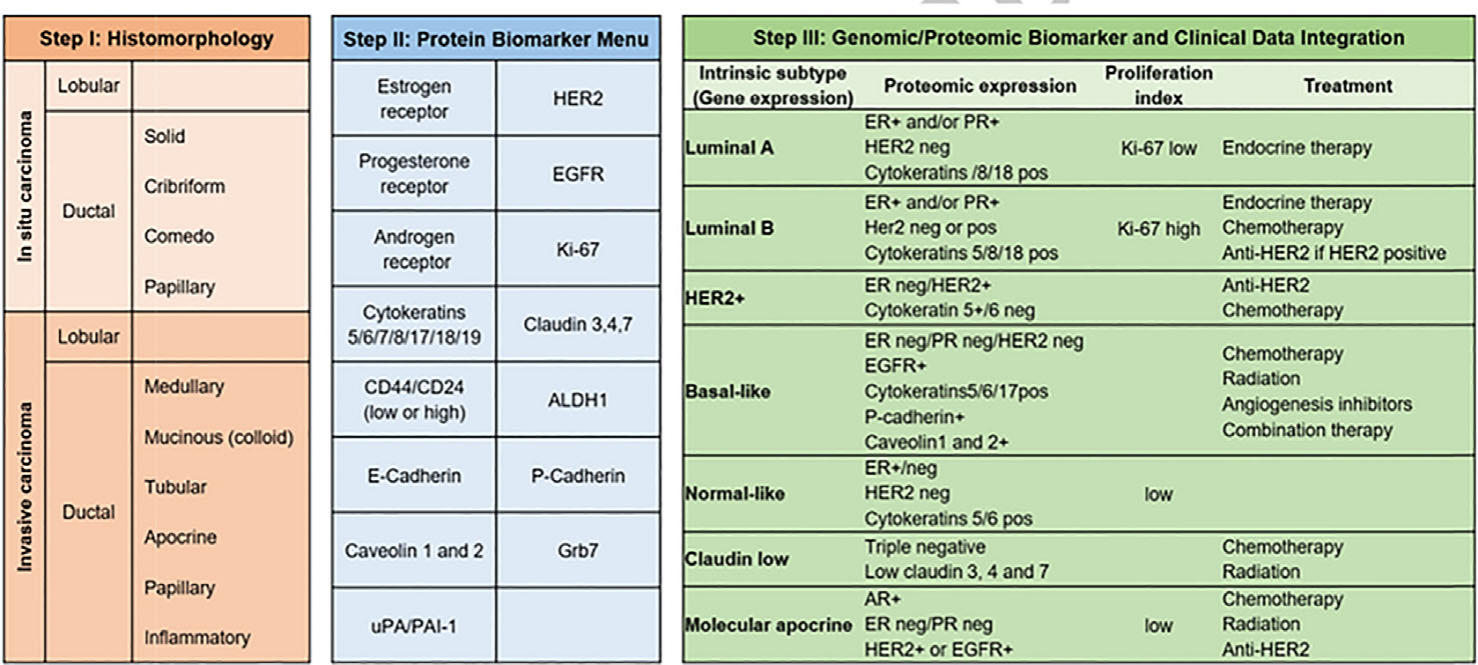
Breast cancer subtypes. From morphology, to immunohistochemistry and transcriptomic classifications (106), breast cancer subtypes have also a deep influence on therapeutic strategy. As reflected in the figure, Luminal A tumors will be treated with endocrine therapy and have the best prognosis among all subtypes, whereas triple negative cancers have the worst prognosis.

Transcriptomics is also essential in breast cancer, where more than 15 years ago, profiling of breast tumors revealed a gene set whose expression varied significantly between tumors 500 gene set revealed 5 gene expression profiles, which were labeled as luminal A, luminal B, basal-like, HER2+, and normal-like, a classification that is used to this day [**Figure 4** - (106)]. This classification has been also relevant for treatment guidance in early-stage breast cancer (107), to predict response to immune checkpoint blockade therapy (108, 109) and to prognosis (110, 111).

#### Small RNAs

Moreover, transcriptomic studies are not restricted to mRNAs that are protein-coding. Other sources of RNA molecules, such as micro RNAs (miRNAs) and long non-coding RNAs (lncRNAs) have also been studied extensively in the context of tumor profiling. For instance, it is well known that miRNAs are widely dysregulated in cancers, and that they may also act as signaling molecules between cancer cells and others in the tumor microenvironment (22, 62, 112), whereas lncRNAs have been involved in cancer immunity, cancer metabolism and metastasis (113, 114). Small RNAs have some advantages over protein-coding RNAs: they are more stable (which is ideal for use in formalin fixed specimens); they can be routinely assessed sensitively and accurately with high-throughput technologies; they can be used as proxies for the mutational status of known, and possibly unknown gene drivers (115); and they may also be assessed in a circulatory setting (as we will explain later for liquid biopsy approaches). Hence, they have been helpful as a source for biomarkers for cancer risk stratification (116), outcome prediction (117) and classification of histological subtypes (118, 119). Nevertheless, their potential is yet to be fully exploited in the years to come, to establish reliable biomarkers to monitor treatment response and guidance of therapeutic strategies.

### Proteomics

Proteins represent the clear majority of therapeutic drug targets in cancer This is due to the fact that many driver mutations result in the structural modification of proteins, and the fact that these particles can also act as signaling molecules with other cell types in the tumor microenvironment (120, 121). Also, the fact that global transcript levels may poorly reflect global protein-level data, makes it of obvious importance to study the changes in proteins occurring in tumor cells that can drive carcinogenesis and influence disease phenotype, aggressiveness, immune escape, and therapeutic response (122).

Thus, the novel proteomic methods that have evolved in the past few years (imaging mass spectrometry, single-cell proteomics, preanalytical sample processing, such as laser microdissection), could be uniquely positioned for the study of cellular populations beyond tumor cells themselves (123). High-throughput proteomic assessments from reverse‐phase protein arrays (RPPA) have been undertaken by TCGA and others in >4000 tumor samples across 11 cancer types (124). Additionally, more comprehensive unbiased approaches using liquid chromatography–tandem mass spectrometry (LC–MS/MS) quantitative analyses have also been performed in the past few years (125–127). These technologies have the potential to identify molecular subtypes and associated pathway features that might be otherwise missed using (epi)genomics and transcriptomics, and indeed have been able to describe ten pan-cancer subtypes across tumor lineages (128). Proteomic studies have also produced significant advances on drug target identification and therapy management, including the observation that treatment with avasimibe, an inhibitor of SOAT1, markedly reduced the size of early-stage hepatocellular carcinomas (129).

### Tumor Microenvironment

Apart from tumor cells, several other cell types are essentially relevant to cancer development and behavior. Potential targets in the TME include the extracellular matrix, cancer-associated fibroblasts (CAFs), immune cells such as infiltrating T-cells (TILs) and macrophages (TAMs). These cells participate in a variety of processes related to tumorigenesis, from immune evasion to angiogenesis promotion (130–132). Indeed, current therapeutics such as bevacizumab already target the TME specifically by inhibiting neoangiogenesis, and the reactivation of lymphocytes is the basis for current immunotherapeutic approaches.

A positive correlation has been observed between the increasing number of diverse cellular subpopulations and patient outcome, which greatly impacts on disease prognosis. For instance, type, density and location of infiltrating T-cells within colorectal tumors can greatly predict disease outcome (133, 134), and the presence of a dense stromal compartment around pancreatic tumors dramatically affects antitumoral therapies in pancreatic tumors (135). One advantage of therapies targeting the microenvironment is that these non-tumor cells are presumably genetically stable, which is in contrast to tumor cells that can accumulate adaptive mutations and rapidly acquire drug resistance (136).

Furthermore, apart from analyzing the heterogeneous contribution of each cell type to a tumor, these extrinsic cell types are also susceptible to profiling by omics approaches that can help refine current therapeutic approaches. For instance, TME profiling may be useful to sub-stratify tumor types classified by other omics (137). Additionally, omics such as RNA-seq or methylation can implement tissue deconvolution strategies that can quantify the contribution of each cell type to the tumor phenotype, and hence help identify novel therapeutic pathways.

### Microbiome

The human microbiome community has fundamental implications in health and disease. In cancer, it has been estimated that 20% of tumors worldwide are microbially driven (138). This includes examples such as HPV-related cervical, oropharyngeal and anal cancers, or *Helicobacter pilorii*-resultant gastric carcinomas, but actually the interaction between the gut normal microbiome and cancer is far more complicated (139). Some cancers appear to be critically dependent on their resident microbiome to continue to subsist and evade the immune system, as in the case of *Fusiobacterium nucleatum* in colorectal cancer (122). This and other studies have revealed causal mechanisms for both microbes within tumors and microbes in other host niches, that can mediate tumor growth through direct and immunological mechanisms (140, 141).

Furthermore, the gut microbiota can define key aspects of drug metabolism, pharmacokinetics, effect and toxicity, since the rate of absorption and bioavailability of many oral drugs depends on their exposure in the gut to both host and bacterial enzymes before entering the circulation. The microbiota can also regulate inflammation and adaptive immunity responses, which in turn can affect cancer immune therapies (142), and tumor profiling has shown to date that distinct gut microbiome patterns associate with consensus molecular subtypes of colorectal cancer (143).

As for examples on the influence of the microbiome in therapy outcomes, mice harboring members of the *Gammaproteobacteria* family in their gut have restored therapeutic effect of gembitacine if concomitant antibiotic ciprofloxacin was administered during treatment (144), and CTLA-4 blockade has been shown to depend on microbiomic response in murine isograft models (145). Hence, the application of novel omic technologies can be very useful in assessing the relevance of these interactions, and the possibilities to address better therapeutic and prognostic value may inevitably pass through microbiome profiling (146).

### Pan-Omics, Big Data, and Data Integration

Clearly, the advantages offered by novel omic approaches are large, and will surely have important repercussions in the development of novel therapeutics in the future. Surely, the availability of these technologies and its affordable costs have also resulted in relevant efforts to combine the different data sources. Emerging systems for better data integration (including *Big Data* approaches) have been focused on filling the gap between generating large volumes of data and our understanding of biology to reproduce the complexity within biological systems.

For instance, the PCAWG consortium has produced data on 20,000 samples from 33 tumor types that includes whole-genome sequencing, DNA methylation, mRNA transcriptomics, miRNA and protein arrays. With this comprehensive overview, they have been able to reveal that tumor clustering across these tumor types is defined primarily by cell-of-origin (147). This has important repercussions in that perhaps the molecular similarities among histologically or anatomically related cancer types could be a basis for pan-cancer therapeutic strategies and drug development, instead of our current arbitrary decisions based on location only. The validity of this argument has to some extent already been validated by the FDA-recommendation to indicate immunotherapy on all MSI cancers, regardless of site, and clearly it will suggest future directions for exploiting clinical actionability in therapeutics.

Another mentionable effort includes the large panel of comprehensively characterized human cancer cell lines (Cancer Cell Line Encyclopedia - CCLE). The CCLE characterized 1,072 tumor lines to include genetic, RNA splicing, DNA methylation, histone H3 modification, microRNA expression and reverse-phase protein array data. Downstream from these analyses, they also performed data integration with functional characterizations such as drug-sensitivity, short hairpin RNA knockdown and CRISPR-Cas9 knockouts to reveal potential targets for cancer drugs and associated biomarkers. The data is publicly available and could provide a resource for the acceleration of cancer research using *ex vivo* models (148).

## Efficiency of Targeted Therapies: Response and Resistance

Choice of therapy is usually undertaken at the moment of diagnosis and throughout disease progression. Apart from the histological information available when the tumor is biopsied, there are two very important factors that determine the therapeutic strategy: response and resistance. Both of those could be aided by omic tumor profiling strategies that could determine the behavior of the tumor prior to drug administration, but are also dynamic processes over the course of treatment (particularly as for the development of secondary resistances). Several examples have been already mentioned in the paper with regards to how tumor profiling at diagnosis can help us define the individuals that will best benefit from a given treatment, with the most prominent likely being the administration of anti-EGFR drugs to *KRAS* wild-type only patients in bowel cancer (149). Currently, further interesting studies are being made on patients exhibiting exceptional responses to systemic therapy, that may provide with unprecedented insights into cancer biology and treatment tailoring (150).

The case for resistance, however, is more complicated, as it is a multi-factorial phenomenon: it summarizes the innate and/or acquired ability of cancer cells to evade the effects of chemotherapeutics and is one of the most pressing issues in cancer therapy. Chemotherapy resistance can arise due to several host or tumor-related factors (151). Resistance can arise at the macroscopic level, based on human organ and/or tissue function, particularly ADME (Absorption, Distribution, Metabolism, and Excretion of drugs) proteins, or at the microscopic level: microenvironmental resistance (changes in pH, glucose or oxygen availability, or changes in TME cell-type composition), or as result of evolutionary resistance. Examples for molecular changes associated with the development of resistance are, for instance, the apparition of the *EGFR* T790M resistance mutation in NSCLC (152), c-MET mutations and loss of anti-VEGF agent effectiveness (153), or therapy resistance mediated by lncRNA inhibition (154), and surely the further is known about the biological features of extensive tumor datasets the more clues we will have to the molecular changes underpinning drug response and resistance.

## Liquid Biopsy

One of the main limitations of tumor profiling to guide therapy comes from the fact that the information we may obtain on neoplastic features is strictly limited by our capacity to obtain tumor samples. In clinical practice, tumor biopsies are taken routinely for diagnosis, but depending on tumor location and accessibility, obtaining a sample representative enough is somewhat complicated, invasive (even with patients with good health status) and costly. Moreover, biopsies are usually taken from the primary tumor, whereas samples obtained from the metastases are often scarce and additionally overlooked for the purpose of treatment decisions.

In the past couple of decades, novel approaches have arisen to detect tumor products from bodily fluids, including the blood, urine or saliva. These are the so-called liquid biopsy procedures, and include the analysis of circulating tumor cells (CTCs), circulating tumor nucleic acids (ctDNA and ctRNA) or tumor exosomes, among others (155–157). Liquid biopsies provide with several advantages over classical approaches, mostly springing from the fact that they are much less invasive and more affordable. Firstly, they provide an unbiased overview of the tumor molecular features, because they are *a priori* not dependent on how well the biopsy is taken. Secondly, they can inform on primary tumor as well as on secondary growths. This is quite relevant when current therapeutic strategies focus mostly on the primary tumor, whereas the majority of cancer mortality is derived from the consequences of tumor spreading. The possibility to obtain data from the metastases as well could implicate a shift into how we design therapeutic approaches to treat cancer patients. Thirdly, they can provide with a dynamic view on tumor evolution and behavior, because they can be taken sequentially over the course of disease and treatment (i.e. monitoring minimal residual disease). This means steady information that can guide monitoring and therapeutic strategies and that could potentially improve overall survival rates.

These considerable advantages have started to take over in the clinics, and there are now FDA-validated blood tests to detect *EGFR* mutations as a first approach to NSCLC treatment (158). Moreover, some studies based on blood biomarker detection have shown presence of resistance variants even before relapse was evident by imaging diagnostics in a few cancer types already (159, 160). Additionally, ctDNA sequencing in colorectal and breast cancer patients can allow for the detection of chromosome copy number and structural alterations that are therapeutically relevant (161, 162), or *HER2* amplifications in patients with gastric cancer treated with trastuzumab (163). Moreover, ctDNA sequencing has also been shown to prove invaluable in order to monitor the evolution of *KRAS* of secondary resistance mutations (164).

## Tumor Heterogeneity and Evolution, and Its Impact in Tumor Profiling

As we have mentioned before, NGS technologies have constituted an important leap in our understanding of cancer, particularly in our search for better, more individualized therapies. However, they are not exempt of limitations, some of which we have already mentioned in the previous sections. There is also another factor to take into account that has considerable impact on treatment response, and that derives from the intrinsic and heterogeneous features of the tumor: clonality and evolution of the tumor. Heterogeneity obviously has great impact on treatment decision and disease progression (165). This evolution is marked by the competition of different clones that arouse from the original cell and acquired the necessary driver mutations to fulfill the hallmarks of cancer and gave rise to the tumor. In this situation, the different clones compete against each other for resources such as nutrients and oxygen, and this provokes an accelerated evolution in terms of the tumor’s intrinsic diversity and heterogeneity (166).

**Figure 5:**
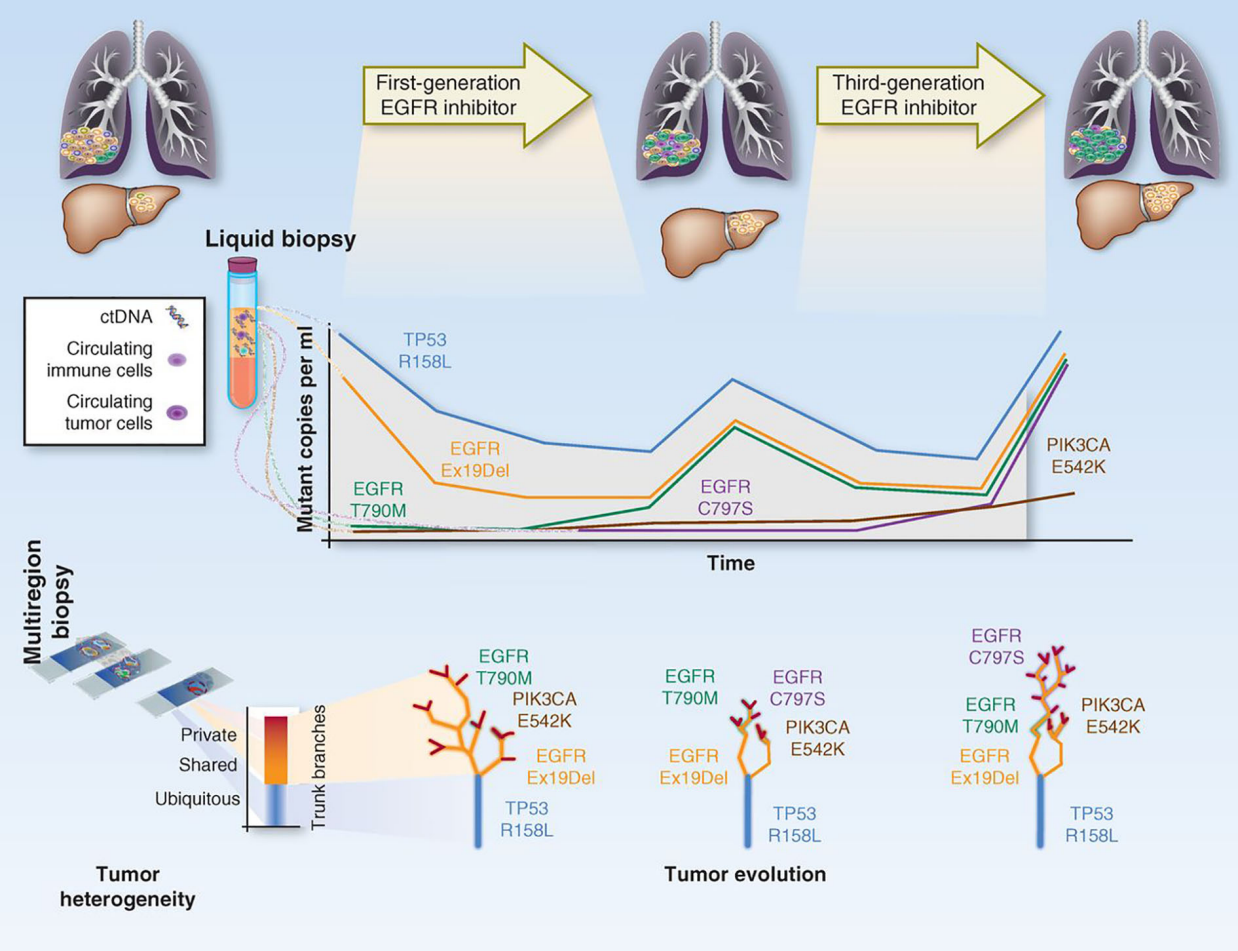
Depiction on how tumor clonal evolution can be integrated into therapy. The figure represents how the tracking of the different clones/mutations can be used to predict treatment response and guide therapeutic decisions. Adapted from (173).

The first implication for this delicately balanced environment is that, when defining targeted therapies for treatment, we have to take into account the clonality of the targeted mutations. At the moment, treatment decisions are based on the rough presence/absence of the mutation. This relies on the sensitivity of the technologies used for detection, but no formal studies have been made to determine if, for instance, the same responses and increases in survival are observed with patients with 1% or 30% mutated allele fractions for a given variant. Because current NGS technologies can give us a more quantifiable overview of how representative a mutation is within a tumor, trials should be designed to define the actionability level of the detected mutations (what is the optimal percentage rate of a mutation in order for it to produce observable endpoint results)?. Secondly, treatment administration profoundly changes the tumor clonality landscape. In other words, when a patient starts therapy, the tumor suffers from accelerated selective pressure, which significantly changes the fitness of each clone and drives heterogeneity variations. This may eradicate major clones that are sensitive, thereby leaving the chance for other opportunistic, and perhaps previously less adaptive clones to flourish, whereas novel mutations may also arise to overcome sensitivity to treatment. This situation applies to all types of chemotherapy, but even more so to targeted treatments, which may well contribute to vanish the responsive clone population at first instance, but may result in the development of resistance once the targeted clones disappear. This phenomenon has been studied extensively by evolutionary geneticists (167–170), and some models have already been produced by liquid biopsy approaches, where each tumor is treated sequentially depending on the dynamic clone proportions that arise over treatment (171, 172) [**Figure 5** - (173)].

## Future Perspectives and the Road to Clinical Implementation

Tumor profiling has the potential to radically change the way we treat cancer. Novel omic technologies, as we have shown in this review, have so far provided us with a considerable amount of information, some of which has already been translated into more efficient therapeutic strategies. Nevertheless, there is a lot of potential to improve and there are several fronts in which profiling could considerably advance therapeutic management and outcome.

For instance, there has already been a shift between classical therapeutic designs decided based on organ and histology toward treatments based on molecular features: immunotherapy is now indicated for all MSI or TMB-high (>=10mutations/Mb) cancers regardless of location, and patients with BRCA-positive tumors of the breast, ovary, prostate and pancreas are treated with PARP inhibitors (35). Because therapy will tend toward a location-agnostic approach, so should diagnostic procedures also. NGS pan-cancer panels have shown to be efficient in detecting actionable mutations in up to 50% of the patients, while allowing for a higher throughput and a quicker turnover than one-by-one IHC approaches (174). Additionally, as often happens with research, although we are at a point where the technologies are vastly available, our capacity to interpret the vast amount of data is limited and overwhelming, and this often results in scientific production failing to translate into clinical practice. Some tools to mine the genomic available info (such as cBio Portal, TCGA database) are already in place, and some will surely become available in the next few years that could help us interpret and integrate the results obtained by omic profiling (175, 176). Moreover, most of the studies on profiling of tumors have been done *a posteriori*, which has given us a lot of insight about tumor molecular features, but may be insufficient to target patients prospectively under the limitations imposed by sample retrieval in the actual clinics. Comprehensive efforts need to be made to protocolize molecular profiling procedures and utilize the data to design meaningful clinical trials that can fill the last step toward clinical implementation of these profiles. Some of these trials have already started to happen, showing great promise in their outcome (177). Another important field of development will be that of broad-spectrum and combined treatments. Synergistic approaches where more than one cancer hallmark is targeted as indicated by the tumor’s own features (anti-angiogenetic factors + different mutation-specific treatments, immunotherapy…) will be key to subdue tumor growth and allow for a better patient prognosis (178).

Overall, cancer molecular profiling will surely revolutionize the way we understand, manage and produce drugs for cancer treatment, and it will be an invaluable tool toward our goal of precision medicine and personalized medicine approaches that guarantee increased patient survival in the near future.

## Author Contributions

CF-R, AS, ML, ÁC, and AC participated in the drafting and revision of this review. All authors contributed to the article and approved the submitted version.

## Funding

This work was funded by AECC grant GC16173720CARR to ÁC, ML, and AC. Also, other sources of funding include the Fundación Ramón Areces; the Spanish Ministry of Science, Innovation and Universities (MCIU- I+D+i 2018), Spanish Research State Agency (AEI), the European Fund for Regional Development (MCIU/AEI/FEDER-UE): grants RTI2018-097455-B-I00 and RED2018-102723-T; CIBER-Onc (grant number CB16/12/00275) and the Economy, Business and Universities Council of the Junta de Andalucia (grant number P18-RT-2501)—to AC; and the Instituto de Salud Carlos III (ISCIII) co-financed by the European Regional Fund (ERDF): grant numbers PI16/01057—to AC and PI15/01262 and CP03/00101—to ML.

## Conflict of Interest

The authors declare that the research was conducted in the absence of any commercial or financial relationships that could be construed as a potential conflict of interest.
